# Disease and Suicide

**Published:** 1859-09

**Authors:** 


// HALL'S JOURNAL OF HEALTH.

OUR LEGITIMATE SCOPE IS ALMOST BOUNDLESS: FOR WHATEVER BEGETS PLEASURABLE AND HARMLESS FEELINGS, PROMOTES HEALTH; AND WHATEVER INDUCES DISAGREEABLE SENSATIONS, ENGENDERS DISEASE.

*We aim to show how Disease may be avoided, and that it is best, when sickness comes, to take no Medicine without consulting an educated Physician.*

VOL. VI.] SEPTEMBER, 1859. [No. 9.

### DISEASE AND SUICIDE.

HUGH MILLER, one of the brightest lights of the nineteenth century, perished by his own hand. He ought to have lived for many years to come, to have enlightened and pleased the world by his writings. Having worked in the stone quarries for fifteen years as a common laborer, a strength of constitution and a capability of endurance were secured to him, which of themselves tended to a long life. In addition, his bodily frame was made to last. He was of medium height, and unusually broad shouldered. An immense head on a long body, with very short legs, gave him a most ungainly appearance, which was not improved by immense tufts of red hair, and queerly notched whiskers. He had so broad a brogue that many of his own countrymen were sometimes puzzled to understand him. But the great mind of Scotland went prematurely out. His life was a life of glory, but he went down to the grave in a cloud. Both continents, in the person of their men of learning, science and religion, with one accord yielded a willing homage to the stone quarrier of Comarty. But notwithstanding all that, "he died as the fool dieth," not in the calmness of christian serenity, but in a kind of *delirium tremens*, in a storm of devils of his own imagining.

*If these things are done in the green tree, what shall be done in the dry?* If men of might, of mind, and of strong religious principle, after a quarter of a century of active and notable usefulness, make their bed in such a hell of mind as Hugh

Miller did, what guarantee have you or I reader that we too may not meet so sad a fate?

This suicide has struck the civilized world with amazement; let us turn so loud a lesson to some useful account. What brought him to the verge of self-destruction? What impelled him to leap into the fearful chasm? If we can find out, then may we avoid the causes and escape the terrible end. The only true biographer is God. He chronicles all the striking points with a sternly impartial pen. Hugh Miller will in time be glorified according to the prevailing fashion. But let us gather passing facts for use while we may; else time will obliterate them, and the world lose the benefit of them. All whole facts are of large value.

Mr. Miller entered on literary life in good health and with a rugged constitution; he lost them both, and died ingloriously—BY LARGE SUPPERS AND LATE HOURS—that tells the whole story. He ate much, exercised little, slept less. These things persevered in, will kill any man. "He always made his supper the principal meal of the day," is the record made of him. "He sat up till the morning hours, straining his mental faculties to the highest possible point," and with a brain thus on fire, laid down, not to sleep, but to troubled dreams and horrid visions ot the night. The morning tound him unrefreshed, haggard and uneasy; and with such a mind and such a body, he girded himself for another day's work. Under such circumstances, the body and the brain were thrown into a state of "*irritation*," medically speaking, in consequence of which, the body was not rested by repose, the mind not calmed by sleep, until at length the power of sleep was lost, when madness came, *and always will!*

In previous numbers of the *Journal*, we have repeatedly advised persons that inability to sleep was a state of great danger; that it preceded insanity, and that it was imperative under the circumstances that every thing like business or study should be instantly abandoned, at whatever sacrifice. For six or seven years Mr. Miller had been conscious of the deleterious effects which continued efforts and anxieties were exerting on his own mind; that they left him only half a man; and to use his own words, "*he could do only half work with dou-*

*ble toil.*" When he engaged in literary employments, he worked with laborious special preparation, with throes which tortured him during the process and then left him exhausted, the victim of nervous depression and irritation. At one time he would sink into the deepest despondency, at another he would be the subject of terrific apprehensions. A friend once found him on the street armed like a bandit, and came very near being shot down. He usually slept with a loaded revolver at his pillow and a broad-bladed dagger near by, while at the head of his bed stood a huge claymore. He lived in such dread of the midnight robber, that these apprehensions became fixed, day and night, asleep or awake; and so vivid did they become, that on the last night of his existence, they drove him from his bed in horror, and after writing the following note to his wife, he sent a bullet through his heart.

" Dearest Lydia.

My brain burns. I must have walked; and a fearful dream rises upon me. I cannot bear the horrible thought. God and Father of the Lord Jesus Christ have mercy upon me. Dearest Lydia, dear children farewell. My brain burns as the recollections grow. My dear wife, farewell.

HUGH MILLER."

What made a wreck of this great mind? ninety-nine and a half out of a hundred will say it was "hard study," "close application," "over mental effort," "excessive working of the brain." This is the judgment of charity: and the cloak of pity and commisseration is thrown over the whole transaction. But for the sake of avoiding so terrific a fate ourselves, let us look into some of the facts of the case in the light of acknowledged physiological truths, sending mere theory to the winds.

The change from a life of bodily labor to one that is sedentary is always attended with danger, and is seldom made with impunity. Under circumstances of daily out-door toil, the appetite for food acquires a *momentum* which carries it into weeks and months of student life. That is, the appetite for food is greater than the actual wants of the system. The person has been eating every day, laying in a store of aliment to repair its wastes and meet the next day's labor; but the next day's labor was left off, and this store was not used. Thus, for a succession of days, the supply exceeds the demand. The instincts of the body, more sleepless than any earthly watchman's eye, exert all the energies of the constitution to work off this surplus, precisely as the faithful engineer keeps the machinery in motion to work off accumulating steam, although the vessel has ceased to move, otherwise an explosion is inevitable. But suppose fire and water were steadily added, and the engine still kept in motion, the machinery would eventually wear out in the natural course of events, and perhaps sooner than if it worked to purpose.

Thus it is in feeding a healthy body for purposes of daily labor. But suddenly ceasing the labor, yet feeding the body still, to the same extent as before, the powers of the constitution are exerted in working off the surplus nutriment; and if kept at it, will as certainly wear out as in mechanics.

In the case of the bodily powers wearing themselves out in this useless labor, several interesting phenomena are presented. The changes are more or less gradual. The stomach first begins to give out, as the brunt of the labor falls first on it.—It is called to digest thrice a day much more than is needed; for a while it does its work, but soon begins to flag; that moment its work is imperfectly done, the material which it supplies, out of which the blood is to be made, is imperfect, and hence imperfect blood is an inevitable result. This blood nourishes the brain, out of it the brain is repaired, out of it the brain grows, out of it the brain is made, and as inevitably must become imperfect, or as we say, "*diseased*," by which we mean that it is unnatural in size, in condition, in function; either larger or smaller in bulk or weight than it ought to be, or of constituents not common to it in a healthful state. The brain itself, the organ of thought, being diseased, the results of its operations, that is the thoughts and the actions of the man must be diseased. Hence we said in a previous number that a dyspeptic man was fit to do nothing right, or more correctly speaking, a dyspeptic is unfit for any thing, for any public station, whether the pulpit, press or bench. Doctors hav'nt time to become dyspeptic, or if they have time, they hav'nt any thing to get dyspeptic on; besides, they have too much sense to precipitate themselves into a disease which make such fools of men.

There is a *delirium tremens* of over eating as well as of over drinking. A penalty is attached to gormandizing alike to that of drunkenness, although public opinion fondles the glutton and throws around him mild charity's most capacious mantle. But nature, the impartial judge, inflicts on both a like penalty. The terrible feature in *delirium tremens* is a horror of imaginary foes, of serpents, dogs and devils pursuing with relentless fury, or torturing with remorseless hate. Not much different is it with an over eater—a dyspeptic. Imaginary terrors seize hold of them, fearful, horrible thoughts invade them. A gentleman suffering from aggravated dyspepsia once said to us " *Doctor, I would be ashamed to tell what thoughts I have sometimes. I am afraid of myself.*"

The day before his death, Mr. Miller said to his physician, " my brain is giving way. I have had a dreadful night of it. I cannot face another such. I was impressed with the idea that my museum was attacked by robbers, and that I had got up, put on my clothes, and gone out with a loaded pistol to shoot them; when I awoke in the morning I was trembling all over."

A kind of nightmare had for some nights rendered sleep most miserable. A sense of vague and intense horror, with a conviction of being abroad in the night wind and dragged through places as if by some invisible power! " Last night I felt as if I had been ridden by a witch for fifty miles, and rose far more weary in mind and body than when I lay down."

Such terrible things were not peculiar to the night, when darkness adds new horrors to a sufferer. He was alone in the dining room in the day time, when a servant entered to spread the table. His face presented such a picture of horror that she shrunk in terror from the sight. He flung himself on the sofa and buried his head in it as if in an agony.

' His suppers were the principal meal of the day !" " Hours after midnight the light was seen to glimmer through his study window." These last two statements were made in the " *Witness*," of which Mr. Miller had been the editor, a day or two after his death; and are no doubt literally correct. Under such circumstances, it may be truthfully said of him—

" *Died of hearty suppers and late hours.*"

And let all public men learn a life-saving lesson from the sad fate of the gifted, lamented and immortal Hugh Miller.

In remonstrating with public men against certain habits of life, we have been often met with the reply, which to the utterer seemed perfectly conclusive—"I am obliged to. I can't help it." Clergymen often feel thus, and sincerely too. Their whole hearts are in their work, and their great anxiety to have things go on smoothly, without a jar, often throws them into positions which seem to make their exposure imperative; positions into which they never would have been thrown, had there not been slackness on the part of others. To such we say—you are of *some* use now at all events, what account will you be if you are dead? The church needs you now. "*A living dog is better than a dead lion.*" The great Head of the Church is not so poorly off, that he needs the sacrifice of your life to carry on the interests of His kingdom. To our mind it borders on extreme vanity; is not short of high presumption for any man to suppose that the Almighty calls upon him to run the imminent risk of his life, let alone its sacrifice, to promote His interests. It would be more becoming to feel "He can do better without me than with me." The best men often feel that they are nothing more than "*cumberers of the ground;*" in other words, that they are more in the way than any thing else.

There is an amount of ignorance, recklessness and presumption on the part of clergymen sometimes, which demands unsparing rebuke, as the result is loss of life occasionally, and very often the loss of valuable time, of months, years, and even the remainder of a long life. Look at the list of invalided clergymen among all denominations, going from the high office of ambassador from the King of Kings, down to vulgar money making by teaching school, editing a religious newspaper, traveling with some rich man's son as tutor, becoming land brokers, penny-a-liners, and all that. We mean no disrespect to these callings, but we do mean to say that it is a degradation for one who has been a minister of the gospel to follow any other calling. We know that it is a necessity in some instances, a pecuniary necessity; ill health making it impracticable for them to pursue their more legitimate business; but the point we aim at is simply this—how came they to be disabled by ill health? There is nothing in severe study of itself to engender disease; as witness the fact that some of the most inveterate students in this and other countries have good health through it all. Multitudes of names could be given of the most voluminous authors, who, beyond the seventies, lived in comparative health, with remarkable vigor of body and mind; such as Humboldt, and Nott, and Silliman, and Benton and others, whose names the intelligent reader will readily recall. We know that as to the private lives of these men, and their compeers in science and literature, and politics, and religion, they were marked by temperance, moderation, system in all things.

Thousands of men of equal mind and genius and promise with those we have named, never lived to meet the expectations of their friends and country; they went to their graves under the forties, the victims of gluttony or drunkenness, benevolently styled in their obituaries, heart disease, dyspepsia, neuralgia, sore throat and the like. Or, if instead of the dyspepsia, spending its force in *sick head ache*, or *neuralgia*, or *gout*, or *sore throat*, or *chronic diarrhœa*, it vents its violence on the brain, as in the case of Hugh Miller, and ends in suicide—partial friends and sympathising editors with one consent begin to make excuse saying—(see *New York Observer*, of May, 1856, in an article headed, "*Self-killing not self-murder*," suppressing names, italics our own.)

"Self-Killing Not Self-Murder.—The late death of ——— Esq., in New York, by his own hand, under a derangement produced by too severe and unrelieved mental labor, has elicited the following feeling tribute from his brother, Rev. Dr. ———, who, at the same time, shows that self-killing is not suicide, or self-murder in this case, because not done in a responsible state of mind. He also draws a moral, with regard to severe mental labor, especially among the young, which is worthy of reflection:

"*To the Editors of the New York Observer:*

"I have a duty to perform to the memory of a deceased brother It is a sad tale that I have to relate of the dead, but it is instructive and admonitory to the living. He was a counsellor at law in the city of New York, and never followed any other business than his profession. In early life, he nearly fell a victim to *intense mental application*. He was considered by his teachers a prodigy of learning when a mere child; and when he quitted Yale College, he had made varied and extensive acquisitions, such as is not common for young men at his age to make. He entered upon the practice of law with the same thirst of knowledge, and became learned in his profession.

"He was an elegant scholar of refined taste, and had a mind amply stored with varied knowledge. He loved the study of botany, and collected and classified almost all the plants of our country; and he was devoted to music. But the cultivation of his taste was ever held subject to the advancement in his profession. He was pure and irreproachable in his character, blameless in his life, retiring in his habits, and free from any moral blemish. He lived respected and loved by all who knew him. *But severe toil in his profession broke down his health. He scarcely took any relaxation for three years past.* Enjoined by his physician, he recently went to Florida and botanized near the Everglades. He returned a week since, not benefitted. His physician feared that fatal disease was upon him. His brain was affected it was thought, with a malady which is incurable, and which induces *paroxysms of mental horror, anguish and despair.* At other times he could attend to his ordinary business. He was in my house during several of those paroxysms before he went south, and which arose, it was apprehended, from a tendency to softening of the brain.

"On Sabbath last there were visible unmistakable evidences of mental aberration. *He ought not to have been left alone.* In the night he arose from his bed, and was heard hastily pacing the floor, and while in a paroxysm of his disease his eye lighted on a weapon which he had purchased for his journey into the wild woods of Florida, he seized it, and instantly terminated his own life. His physician, one of the most able in his profession in the city, has written me as follows: After narrating the means he had recommended for his benefit, he says, 'the malady seeming to be evidently on the increase, it was intended to propose means for careful watching on any marked mental derangement. Such derangement appeared sooner than was anticipated; and while under the influence of a paroxysm of such derangement, and evidently without its being premeditated, he was *permitted by an inscrutable Providence* to be the agent of his own destruction.'

"A coroner's inquest hastily assembled, without any proper inquiry into the state of his mind, and, without summoning his attending physician, or his friends, gave an inquest of 'suicide from a pistol shot.' And it is now published over the country and the world, that my brother committed suicide. What a strange misnomer! 'Suicide is self-murder, the act of designedly destroying one's life.' 'To commit suicide' says Blackstone, 'one must be of years of discretion and sound mind.' This is the only definition of the term. My brother, when he died, was not of sound mind. He never contemplated, nor committed suicide. His act had no attribute of this crime, because it proceeded not from a sound mind, but one diseased and utterly unhinged from its responsibility. Self-killing is not self-murder. There was no act of a conscious and intelligent self involved in the case. If my brother had shot the whole family, it would not have been murder. In his state of mind he could do no responsible act; and he was not guilty of self-murder. As well might it be claimed that one is responsible for being struck with lightning; or for killing himself by falling in a fit of epilepsy. *The hand of God took away his reason*, and that was the fatal blow.

"While on my way to ———, the place of sepulture, I read the newspaper accounts, and then felt that I had another duty to perform than merely commit my brother to the dust. His friends received his body at the depot, and bore it to the church, where funeral services were performed, and then to the cemetery. This is a lovely spot, on the eminence by the church overlooking the river, and the site of the Palisade Fort, constructed more than two centuries ago. When the coffin had been lowered to its rest, I stood forth, chief mourner that I was, and thanked the assembly for their kind attention; and then narrated to them the history of my brother's life, labors and melancholy death. I told them that I felt it to be due to him, and to the memory of his ancestors, among whose graves we stood, to bear my testimony to his unblemished life, and irreproachable character. He had never disgraced his parentage, nor the place of his birth. He was to rest in a grave amid those who had lived and died without reproach. His dust was to mingle with that of the worthies whom his maternal ancestor, six generations ago, had as their pastor, led through the wilderness to become the first settlers of that town and of the State. His father had for forty-six years ministered in the gospel to this generation, and had succeeded in the pulpit which his father had left vacant. My brother was to be buried by their side. And I could not bear that the citizens of our native town, on retiring from that spot should be told in the public prints that they had done honor to one who is unworthy. I do that intelligent and kind people no injustice when I say, that every heart seemed to respond to the propriety of my remarks, and that if tears give any expression of sympathy, those tears were not withheld.

"There are lessons of wisdom contained in this brief narrative, to parents, students, and men of professional life. Three of my brothers —one a physician, one a clergyman, and another a lawyer—have gone to the grave in the midst of their years, under the severe and unmitigated toil of their professions. It will not do to give the mind no proper relaxation; like a bent bow, it will snap when least expected. Nor should children of precocious intellect be urged on to become prodigies of learning. They should rather be diverted from their studies. Parents are not often aware of the danger. The brother whose untimely death I mourn, had read the Bible through and aloud to his mother, before he was six years old and he had completely mastered the common school arithmteic for advanced scholars at the age of eight. It was this intense application of his useful mind which laid the foundation for his final melancholy end.

"It seems but a few short years since we were all young and in our father's house. We never knew any differences. What one brother possessed all enjoyed. We were thus educated. We would have laid down lives for each other. William was the youngest and the favorite. He was always of a filial disposition, the kindest of brothers, and the most studious and faithful of men. We called him little Willie when a child. He was his mother's companion during the wearisome years of infirmity which preceded her decease. He preferred, with a book in his hand, to remain in her company; and no inticement could induce him to leave her for the society of the children of his age, and to engage in their sports. He would always pluck for her the finest peach in the garden, the prettiest flower which he could gather. I think I see him now, as was his usual custom, seated in his little chair, his Bible laid on another chair before him, and reading aloud to his mother in her sick room. I see the eye of that parent beaming with delight in view of the bright prospect opening before her darling child. Could such filial piety, she thought, ever go unrewarded? That kind mother has long ago passed away, and that loved child now sleeps by her side."

The above letter is full of instruction and warning, and ought not to perish with a newspaper. The editors of the *Observer* join with their distinguished correspondent, the Rev. Dr. ———, in the effort to show that if a man kill himself in a state of mind induced by "*too severe mental labor*" it is not suicide. They aim to shield the memory of their friend from the imputation of "suicide," because a blot attaches itself to such an act the world over, we instinctively shrink away from it. When Hugh Miller perished similarly, the same paper, dated January 22d, 1857, in an article headed "*Overworking of the Brain*," page 26, says—

"And so by his own hand, in an hour of delirium, one of the noblest sons of genius and learning and religion has perished. In our regret that in the midst of his days he came to such a melancholy end, we forget every thing in his published writings that might be supposed to *lessen our respect for the man*, while we remember him as the champion and eloquent historian of religious liberty, the pious student of God's out-of-door world, and the friend of human kind.

"We would take his death as a text from which to offer a word of exhortation to ourselves and our friends on the sin and danger of overworking the brain. Hugh Miller was a *victim to this vice.* What is it but a *crime* against God and society, and one's family and one's self, to task and whip and drive the brain to madness? Such cruelty to the limbs we denounce in glowing periods, and seek to draw the scorn of men upon its perpetrators. But men of business will pursue the world with intensity and restlessness, bolting their food in hot haste, planning and pushing twelve, fifteen, twenty hours a day, leaving little time for sleep, none for *repose*, and so it comes to pass that paralysis, consumption, brain fever, derangement cut off so many at the very time of life when they thought to be prepared to rest.

"It is so and worse with men in professional life. They work their brains and nothing else. Hard at it, and always at it, studying, writing, speaking, *dreaming* of the labors of the day when dreaming at all, they make themselves martyrs to their profession and verily think they are pleasing God by their diligence, when they are murdering themselves. To take an infinitesimal pill of poison daily so as to commit suicide in ten years they would shrink from as blood guiltiness. But to strain the nervous system beyond its nature, and thus gradually to undermine and ruin it, is a *crime they commit* in spite of their daily prayer 'So teach us to number our days that we may apply our hearts unto wisdom.'

"This is their folly and their sin. *It was a sin in Hugh Miller to make himself a madman by overwork. It is a sin in any man* no matter what is his business or pursuit, to devote so much time and thought to it as to interfere with the enjoyment of present and future health. The laws of nature are the laws of God. They cannot be violated by rational beings, without sin and punishment. We have examples to warn us, furnished constantly in the circle of our own acquaintance, in the records of our newspapers, and the statistics of our Asylums. 'Therefore let him that thinketh he standeth, take heed lest he fall.'"

The above sentiments are so just and true that they are heartily commended to the mature consideration of all reflecting men.

What were the causes which wrought such a total change in the sentiments of the paper between May of one year, and the succeeding January, it is not important to inquire, let it be laid to the door of progress.

We trust that the points of this article will not be forgotten.

*First*—Suicide, self-killing or "slaying one's self," which is the literal meaning, is a crime against God and man under all conceivable circumstances.

*Second*—It is no extenuation that the victim is mad or crazy or insane at the time, if he brought that condition of mind on himself by any sort of intemperance or self-indulgence whatever.

If a man eats himself mad, or drinks himself mad, or yields himself to any other self-indulgence until madness takes place, and in such madness he murders himself, or somebody else, the murder so committed is a sin against humanity, and he who palliates it, is not himself guiltless.

To all therefore we say, life is a talent. If you hide "it in a napkin," or loose it by ignorance or inattention, or throw it away by over indulgence in any animal appetite or passion, what account will you give to the Master when he makes requisition?

